# Bibliometric analysis of rheumatology research in the Arab countries

**DOI:** 10.1186/s13104-016-2197-x

**Published:** 2016-08-08

**Authors:** Karim Bayoumy, Ross MacDonald, Soha Roger Dargham, Thurayya Arayssi

**Affiliations:** 1 Weill Cornell Medicine - Qatar, Education City, Al-Luqta Street, P.O.Box 24144, Doha, Qatar; 2 Distributed eLibrary, Weill Cornell Medicine - Qatar, Education City, Al-Luqta Street, P.O.Box 24144, Doha, Qatar; 3 Biostatistics, Epidemiology, and Biomathematics Research Core, Weill Cornell Medicine - Qatar, Education City, Al-Luqta Street, P.O.Box 24144, Doha, Qatar; 4 Department of Internal Medicine, Weill Cornell Medicine - Qatar, Education City, Al-Luqta Street, P.O.Box 24144, Doha, Qatar

**Keywords:** Bibliometrics, Scientometrics, Arab countries, Rheumatology, Web of Science

## Abstract

**Background:**

The Arab world has seen an increase in the burden of musculoskeletal diseases. No bibliometric studies have characterized rheumatology research in the Arab world. This study evaluates the productivity and impact of rheumatology research in the Arab world.

**Methods:**

We searched the Web of Science Core Collection for rheumatology publications, from 1976 to 2014, for each of the Arab League (AL) countries, North America, Europe and Asia. For the AL countries, the overall trend of publications and citations was analyzed, while considering the paper type and collaborations.

**Results:**

The AL countries published 944 rheumatology papers over the period studied. The number of publications increased by a factor of 2.77 (95 % CI, 2.75–2.78) each decade, and citations increased by a factor of 2.36 (95 % CI, 0.96–5.82). The absolute number of papers included in the top-10 rheumatology journals remained constant but the proportion decreased. Papers involving collaboration among AL countries were found to increase over time.

**Conclusions:**

Overall, the AL countries lag in research productivity and impact compared to other regions. Three countries are responsible for the majority of publications, while four countries receive the majority of citations.

**Electronic supplementary material:**

The online version of this article (doi:10.1186/s13104-016-2197-x) contains supplementary material, which is available to authorized users.

## Background

The Arab countries comprise the 22 countries that are members of the League of Arab States, whose total population is around 300 million [1]. This region has undergone an epidemiological shift in the past 20 years [2]. The disease burden has shifted from communicable, maternal, nutritional and neonatal diseases to non-communicable diseases; including the emergence of musculoskeletal disorders, which are the 10th and 6th highest contributors to disability adjusted life years (DALY) among all ages and ages 30–59 respectively [2] (Additional file 1: Tables S1A, B). Low back pain, for instance, a common musculoskeletal condition, has become the 7th largest contributor to DALYs in the Arab region.

Despite this change in the burden of disease, locally driven research in general, and in the field of rheumatology in particular, is lagging overall [3]. In a recent study, the Middle East region contributed to 4.46 % of all rheumatology publications between the years 1996 and 2010 but none of the Arab League countries appeared in the top 35 countries participating in this knowledge production [4]. Bibliometric analyses have been useful in interpreting relevant trends in clinical practice and the evolution of research behavior [5]. The purpose of this report is to examine the trends in published rheumatology research in the Arab League (AL) countries. In particular we aim to: (1) Describe trends in number of rheumatology publications produced by the AL countries; (2) Use a strategy combining Medline’s Medical Subject Heading (MeSH) publication type descriptors with Web of Science (WoS) citation counts to determine the number and impact of publication types produced by the AL countries; (3) Examine the trends in the number of AL rheumatology papers that appeared in the top impact factor journals; (4) Assess the influence of collaboration on the impact of AL papers.

## Methods

### Database searching

The Web of Science Core Collection database (WoSCC) was used to find papers for this study. Papers were identified by searching for “Rheumatology” in the Web of Science Category field, i.e. WC = (Rheumatology). Papers from individual countries were then identified using the WoS Country field, e.g. CU = (Algeria), while papers from all the AL countries, were identified by searching for multiple countries e.g. CU = (Algeria OR Bahrain OR…). The CU field tag indexes the country affiliations of all authors, therefore, papers which appear in a specific country search are included if any author has an institutional affiliation in that country. All searches in WoSCC were restricted to the “Article” document type and the years 1976–2014. Details of all searches are shown in Additional file 1: Annex S1.

For each search, the “Analyze Results” function of WoSCC was used to rank results according to publication years, authors, countries/territories (i.e. country affiliations of authors), and source title (i.e. journal), as required. Search results were exported to Microsoft^®^(MS) Excel^®^ for Mac 2011 Version 14.1.0 (Microsoft Corporation, 2010, Redmond, Washington) and further analyzed as noted below.

### Publication type determination

Publication types were defined as per the National Library of Medicine’s (NLM) MeSH database [6]. The document type scheme used in WoSCC [7] was found to be insufficiently granular to assess the output of different kinds of papers. For example, the WoSCC “Article” document type can include case studies or randomized controlled trials (RCTs), among others. In contrast, the MeSH database distinguishes between these study types based on examination of individual article content. Therefore, a separate search of Medline was conducted using WoS to retrieve the rheumatology papers previously found in WoSCC. Exporting the Medline search results into Microsoft^®^ Excel^®^ allowed the NLM publication type to be combined with the citation data from WoSCC.

### Collaborations

Local collaborations were defined as publications that included author affiliation addresses from two or more AL countries. Collaborative papers were identified by their appearance in the result lists of more than one country-specific search. The set of papers retrieved was manually examined for affiliation of the first and senior author, to approximate the number of publications with substantial contribution from AL affiliated authors.

### Determination of the number of Arab League papers appearing in the top ten rheumatology journals

The ten most highly cited rheumatology journals for the period 1997–2013 were determined using the Journal Citation Reports® database: for each year, the “Rheumatology” category was selected, and the resulting list of journals was sorted by Journal Impact Factor to identify the ten journals with the highest impact factors. To identify those papers from Arab League countries appearing in these journals each year, the WoSCC search results previously exported to MS Excel were sorted by year and journal title, and a count was taken of the papers appearing in the top ten journals in each year.

### Statistical analysis

Linear regression was used to analyze trends in publication and citation, with log-transformation performed, when suitable, to ensure that the data followed a normal distribution. Statistical significance of correlations was determined with Spearman and Pearson tests (depending on normality of data). p-Values were reported and tested at the level of significance of 0.05. Statistical analysis was performed using Microsoft® Excel® for Mac 2011 Version 14.1.0 (Microsoft Corporation, 2010, Redmond, Washington) or Stata 13 commercial statistical software (StataCorp, 2011, College Station, Texas).

## Results

### Number and trend of publications

**Fig. 1:**
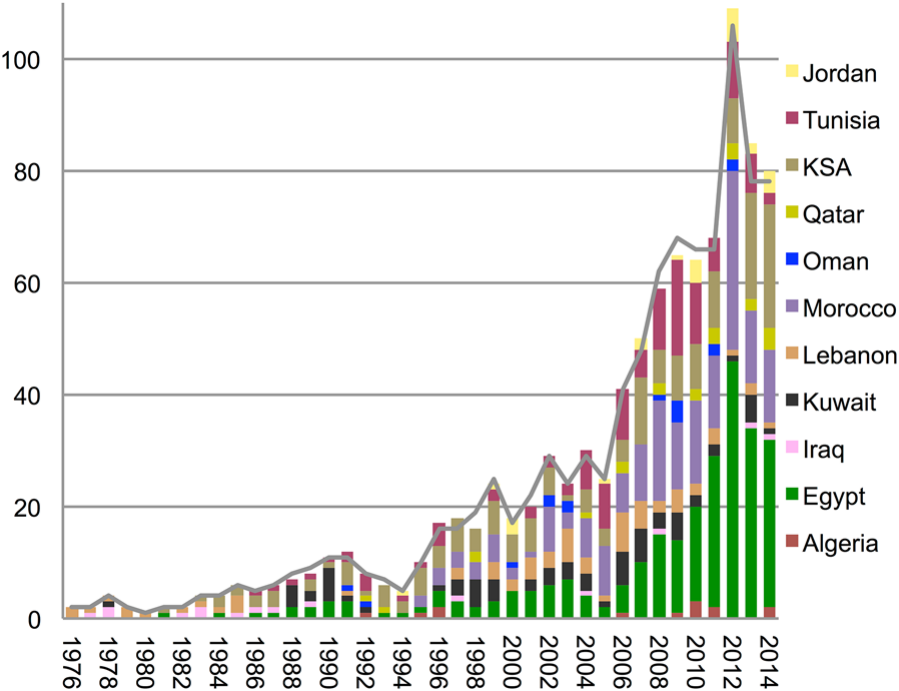
Annual publications for The Arab countries (1976–2014). The *gray line* indicates the total publications. Only countries with more than ten publications have been included. The overall number of publications is N = 944.* KSA* Kingdom of Saudi Arabia

Seventy five thousand nine hundred and five rheumatology papers were retrieved from WoSCC between 1976 and 2014. Nine hundred and forty-four (1.2 %) were published from the Arab League countries, with the first two papers published from Lebanon in 1976 [8, 9]. For every decade since 1976, the number of publications from the Arab League countries steadily increased by a factor of 2.77 (95 % CI 2.75–2.78): 29 publications between 1976 and 1985, 80 between 1986 and 1995, 222 between 1996 and 2005 and 613 between 2006 and 2014 (p < 0.05) (Fig. 1). Egypt (26.91 %), Morocco (18.96 %) and the Kingdom of Saudi Arabia (KSA) (18.11 %) contributed around two-thirds of publications (Fig. 1). No publications were found from Palestine, Djibouti, Comoros, Somalia, or Yemen.

### Publication types

Nine hundred of the 944 rheumatology papers from the Arab League countries were found to have MeSH publication type descriptors [6]. Of these, 231 papers (24.5 %) were described as case reports, 76 (8.1 %) as comparative studies, 31 (3.3 %) as reviews, 24 (2.67 %) as clinical trials, 22 (2.3 %) as multicenter studies, 15 (1.6 %) as randomized controlled trials (RCTs), and 15 (1.6 %) as evaluation studies (Additional file 1: Table S2). From 1976, there was a significant (p < 0.05) increase from decade to decade in comparative studies 15.2 (4.5–25.9) and reviews 6.5 (1.5–11.50) while case reports 41 (−4.21–86.21), multicenter studies 6.2 (−6.77–19.17), RCTs 3.4 (−2.08–8.88) and evaluation studies 4.6 (−5.6–14.80) demonstrated a non-significant positive trend.

Although multiple MeSH descriptors may be applied to a single paper (e.g. a RCT may also be a multicenter study), it is still possible to see trends in the use of publication type (MeSH) descriptors and therefore the type of study being published.

### Citations and impact

**Fig. 2:**
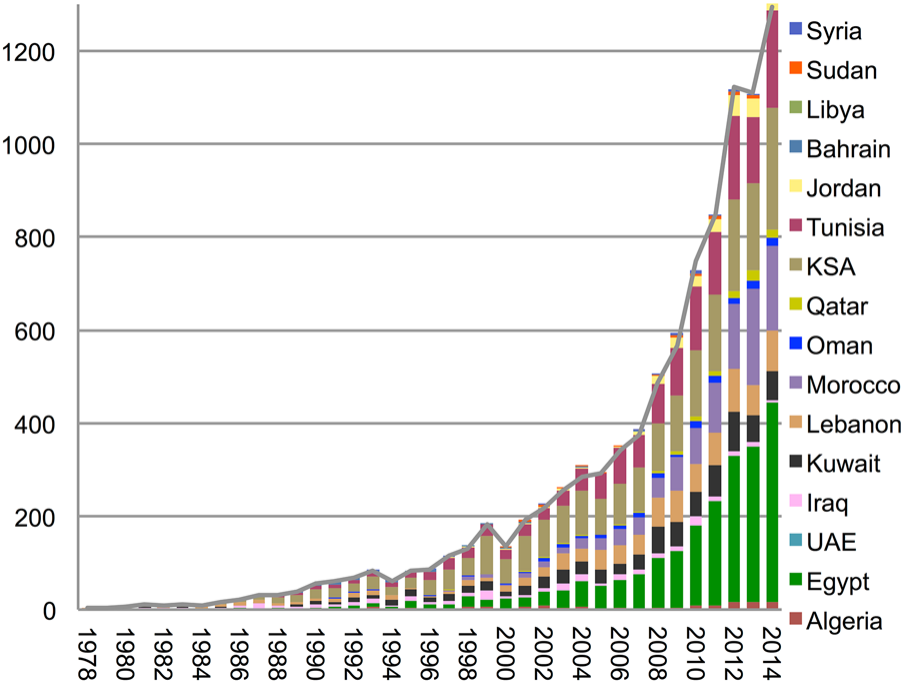
Annual number of citations per year for the Arab league countries (1976–2014). The *gray line* indicates the total number of citations. *KSA* Kingdom of Saudi Arabia, *UAE* United Arab Emirates

Until the end of the year 2013, 866 papers were published, of which 762 (88 %) were cited at least one time by the end of 2014, leaving a total of 103 (11.89 %) non-cited papers by the end of 2014. As of the 31st of December 2014, the region had a total of 9394 citations and average citation per paper of 9.95. The number of citations increased by a factor of 2.36 (95 % CI, 0.96–5.82) from decade to decade, but this was not statistically significant. Approximately two-thirds of the citations were received by publications from KSA, Egypt, Tunisia, Morocco and Lebanon (Fig. 2).

### Impact by publication type

From the set of 944 publications, only 900 papers were associated with a PMID on WoS. A total of 830 were published by the end of 2013, and evaluated for citations until the end of 2014. Randomized controlled trials, multicenter studies, clinical trials and comparative studies garnered the most citations per paper out of each publication type, with average citation per publication of 25.57, 23.94, 20.21 and 16.09 respectively. Reviews, evaluation studies, and case reports had lower average citations per publication with 10.89, 9.92, and 6.24 respectively (Table 1).

**Table 1:** Comparison of average citation per paper between different paper types

Type of publication	Average citation per paper
All papers	12.2
Case reports	6.24
Comparative studies	16.9
Reviews	10.89
Multicenter	23.94
RCT^#^	25.57
Evaluation	9.92
Top ten	22.75
Collaboration between 2 AL countries	11.2
Collaboration between 3 AL countries	19.2

^#^Randomized controlled trial

### Publications in the top impact factor journals

**Fig. 3:**
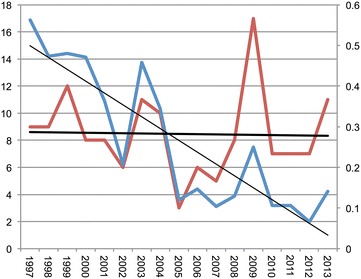
The annual representation of articles in the top ten-rheumatology journals from 1997 to 2013 (by 2 year impact factor in the journal citation index). Absolute number of publications is represented on the *left axis* (*red line* and *thick trendline*). Proportion of papers is represented on the *right axis* (*blue line* and *thin trendline*)

During the period 1997–2013, for which the journal citation reports runs, the number of publications in the top ten rheumatology journals per year has ranged from three to seventeen papers with an average citation per paper of 22.75 [10]. The absolute number of papers included in the top ten rheumatology journals did not change significantly (p > 0.05) over 1997–2013, however, there was a significant decrease in the proportion of papers included in the top ten journals relative to the amount of publications in each respective year (p < 0.01) (Fig. 3).

### Arab collaborations

**Fig. 4:**
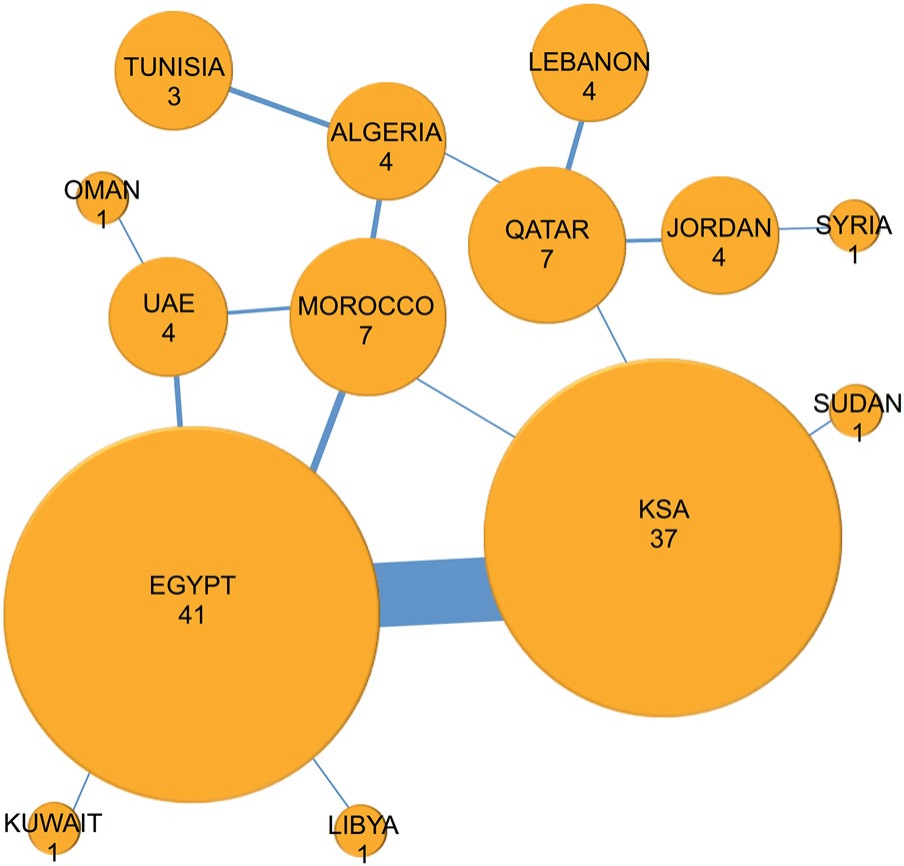
Collaborations among the Arab League countries. The *thickness of the line* is proportionate to the number of collaborative publications. *KSA* Kingdom of Saudi Arabia, *UAE* United Arab Emirates

From the 944 publications, only 55 (5.82 %) involved collaboration between two or more authors from AL countries. Six of these papers included collaboration amongst three AL countries (Fig. 4), which was the maximum number of Arab countries collaborating on a single paper. The first collaborative paper between AL countries was published in 1991, and included KSA and Egypt [11], which appear to have the most overall collaborations in the region (n = 35 out of 55, 63.63 %).

Egypt has the greatest degree of collaboration with other AL countries, both in number of collaborating partners (n = 5) and number of papers resulting from collaboration (n = 41). KSA follows with four partners and thirty-seven papers, then Morocco and Qatar with four collaborating partners and seven papers each.

The list of 55 publications was further manually inspected to determine the country affiliation of the first and senior authors. 11/55 (20 %) were found to have no first or senior author affiliation to an Arab country. 26/55 (49.5 %) were found to have a first or senior author with a dual affiliation in KSA and Egypt. Of the 26 with an affiliation to an Arab country, 5 had both the first and senior author hold a dual KSA and Egypt affiliation.

### Impact of locally collaborative papers

Of the 55 papers involving collaboration between AL countries, complete citation information was available only for the 44, which were published between 1997 and 2013. Fifteen (34 %) of these were published in the top ten ranking rheumatology journals of their year of publication (as measured by journal impact factor). In contrast, of the 697 publications from the same period that did not involve collaborations within the AL countries, 129 (19 %) were published in the top-ranking rheumatology journals. In addition, publications involving a greater number of AL collaborations appeared to have a higher average citation (Table 1). It should be noted however, that the small sample size involved makes it difficult to draw a conclusion as to the significance of this difference.

## Discussion

Our data demonstrates that rheumatology publications from the AL countries still lag behind other world regions. Several factors may influence the underrepresentation of rheumatology research in the region. These may include bias from journals toward publishing articles from their country of origin in favor of articles of local interest, WoS may not include journals from middle to low-income countries [12] or articles may be published in local Arab journals that are not indexed on WoS [13, 14]. In addition, the productivity of a country may be skewed based on its number of indexed journals, which are relatively few in the Arab world [15, 16]. Within the region, however, and over the period of 1976 to 2014, we found a consistent and significant growth in the number of publications. This is not unexpected as the world overall is seeing an increase in rheumatology publications, particularly from emerging economies [4, 17]. The Arab world in particular, has developed significantly over the past three decades, with an improvement in life expectancy and an increase in the burden of non-communicable diseases [2]. It is encouraging to see this growth in research, despite ongoing conflicts in many Arab countries, a high rate of brain drain and limited opportunities for research funding [3, 18].

Despite a changing trend towards the more impactful papers, about one quarter of the publications are case reports that have the lowest average citation per paper as compared to other publication types. Although case reports can be helpful to the education and career advancement of residents and junior academic clinicians, and to understanding rare conditions [19], they rarely contribute to new knowledge and are seldom published in top tier journals, likely explaining the paucity of publications in the top ten rheumatology journals that we have observed. Changing this trend will require training more rheumatologists to meet patient needs and engage in meaningful research. To achieve this, funding agencies must understand the societal impact of musculoskeletal diseases, then develop clinical research capacity and promote collaboration [20].

The trends of publication from Tunisia and the KSA were noted to have unusually dramatic changes. Tunisia was seen to have a rather consistent output of publications from 2004–2010, after which publication volume fell. Coincidentally, the Jasmine revolution, which began at the turn of 2011, destabilized the government and economy of Tunisia [21]. This may have affected research funding and efforts in the country.

We also noted that the KSA had relatively higher publications in 2013 and 2014 compared with their historical performance. This is in line with previous studies showing an increase of publications from KSA, which was attributed to progressively increasing expenditure on education and research [22]. Whether this expenditure is responsible for meaningful research in rheumatology [23], may be difficult to determine due to the overall scarcity of publications.

In our assessment of collaboration using standard country address methods, we noted that apparent collaboration may not be due to coauthors from different countries, but rather due to dual affiliation of a single author in two AL countries. While our analysis of impact by number of collaborations was inconclusive due to the small number of papers, it has been observed that papers involving more collaborators tend to be more impactful [24].

There are several limitations to our study. First, the most significant limitation is the small sample size of papers available for analysis, which affected interpretation of our results. Second, as with any bibliometric analysis, we are dependent on the coverage and indexing practices of the database used. We chose WoSCC as it appears to index all of the major rheumatology journals over a sufficient time period to give a historical perspective on publications in the Arab league countries. In addition, WoSCC’s scheme for classifying publication types was insufficiently granular, which necessitated the use of the Medline scheme. Also, our determination of the countries involved in each paper was based on WoSCC’s indexing of the authors’ addresses; these may reflect institutional affiliation rather than the true geographical location of the author or research [25, 26].

A final limitation in our search was our focus on English-language publications. Many papers from the North African/Maghreb countries are published in French. We may therefore be underrepresenting research productivity from several of the AL countries [3]. Citation rate is a commonly used proxy for the impact of a paper. The data here may underestimate the impact of recent papers, which are still in the process of receiving citations.

## Conclusions

This paper is the first bibliometric analysis of rheumatology research in the Arab League countries. Despite the increase in publications, the Arab League countries lag in rheumatology research compared to other regions and the overall impact of the papers appears to be declining. Three countries are responsible for the majority of publication while the most citations were attributed to only four countries. Future in depth studies of those highly cited authors and institutions, and the effect of collaboration, is warranted to identify best practices that could be shared with others in the region.

## Additional file

10.1186/s13104-016-2197-x Estimated disability adjusted life years (DALY) for non-communicable disease (2012) for all ages. **Table S1B.** Estimated disability adjusted life years (DALY) for non-communicable disease 560 (2012) for ages 30–59. **Table S2.** Raw data based on all paper type categories listed in Medline.** Annex S1.**All searches in the Web of Science Core Collection were restricted by Document Type=(Article) and Timespan=(1975–2014).

## Abbreviations

DALY: disability adjusted life years
AL: Arab League
MeSH: Medical Subject Heading Database
WoS: Web of Science
WoSCC: Web of Science Core Collection
WC: Web of Science Category Field
CU: country field
MS: Microsoft
NLM: National Library of Medicine
RCT: randomized controlled trial
KSA: Kingdom of Saudi Arabia
