# Supplementary material for: Bibliometric analysis of rheumatology research in the Arab countries
Source: BMC Res Notes. 2016 Aug 8;9:393. doi: 10.1186/s13104-016-2197-x (PMC4977706; doi:10.1186/s13104-016-2197-x)
Supplement: Supplementary file 1 — 10.1186/s13104-016-2197-x Estimated disability adjusted life years (DALY) for non-communicable disease (2012) for all ages. Table S1B. Estimated disability adjusted life years (DALY) for non-communicable disease 560 (2012) for ages 30–59. Table S2. Raw data based on all paper type categories listed in Medline. Annex S1.All searches in the Web of Science Core Collection were restricted by Document Type=(Article) and Timespan=(1975–2014). [file 13104_2016_2197_MOESM1_ESM.docx]

**Supplement:**

**Table S1A**

Estimated disability adjusted life years (DALY) for non-communicable disease (2012) for all ages.[1]

| **Disease Category** | **DALY (Total, 000s)** |
| --- | --- |
| Cardiovascular diseases | 17742.2 |
| Infectious and parasitic diseases | 15696.7 |
| Neonatal conditions | 13160.6 |
| Unintentional injuries | 10612.0 |
| Mental and behavioral disorders | 10351.9 |
| Respiratory infections | 9073.8 |
| Malignant neoplasms | 7132.8 |
| Intentional injuries | 6703.1 |
| Nutritional deficiencies | 5292.8 |
| Musculoskeletal diseases | 4782.2 |
| Respiratory diseases | 4150.7 |
| Congenital anomalies | 4031.3 |
| Diabetes mellitus | 3536.5 |
| Digestive diseases | 3383.8 |
| Neurological conditions | 2806.6 |
| Genitourinary diseases | 2757.7 |
| Sense organ diseases | 2630.5 |
| Endocrine, blood, immune disorders | 1819.0 |
| Maternal conditions | 1042.5 |
| Skin diseases | 988.6 |
| Oral conditions | 671.5 |
| Other neoplasms | 382.9 |

**Table S1B**

Estimated disability adjusted life years (DALY) for non-communicable disease (2012) for ages 30-59.[1]

| **Disease Category** | **DALY (total, 000s)** |
| --- | --- |
| Cardiovascular diseases | 6049.1 |
| Mental and behavioral disorders | 4552.6 |
| Unintentional injuries | 3391.2 |
| Malignant neoplasms | 3267.0 |
| Infectious and parasitic diseases | 2927.9 |
| Musculoskeletal diseases | 2539.9 |
| Intentional injuries | 2236.6 |
| Digestive diseases | 1594.3 |
| Diabetes mellitus | 1570.2 |
| Respiratory diseases | 1377.6 |
| Genitourinary diseases | 1228.0 |
| Respiratory infections | 1177.6 |
| Neurological conditions | 907.0 |
| Nutritional deficiencies | 902.8 |
| Sense organ diseases | 868.7 |
| Maternal conditions | 487.3 |
| Endocrine, blood, immune disorders | 421.4 |
| Oral conditions | 341.7 |
| Skin diseases | 319.4 |
| Other neoplasms | 194.7 |
| Congenital anomalies | 141.3 |
| Neonatal conditions | 124.2 |

**Table S2:**

Raw data based on all paper type categories listed in Medline.

| **Publication Type** | **Average citations per paper** | **Number of Publications** |
| --- | --- | --- |
| Multicenter Study | 23.94 | 17 |
| Case Reports | 6.24 | 226 |
| Validation Studies | 18.81 | 16 |
| Comparative Study | 16.09 | 74 |
| Observational Study | 0.00 | 0 |
| Randomized Controlled Trial | 25.57 | 14 |
| Evaluation Studies | 9.923 | 13 |
| Review | 10.90 | 29 |
| Clinical Trial | 20.22 | 23 |
| Controlled Clinical Trial | 12.00 | 6 |

No papers were omitted in this analysis due to empty field or otherwise. Based on 830 publications until 2013 and citations until the end of 2014.

**Annex S1:**

All searches in the Web of Science Core Collection were restricted by Document Type=(Article) and Timespan=(1975-2014).

Arab league[2]:

The following search was performed for each country:

WC=(Rheumatology) AND CU=(country name). Individual searches were combined with “OR” to retrieve all papers published in the Arab league.

[1] WHO | Global Health Estimates 2015. Available from: <http://www.who.int/healthinfo/global_burden_disease/en/>. Accessed 2 July 2015.

[2] Official Arab League Website. Available from: <http://www.lasportal.org/Pages/Welcome.aspx>. Accessed 3 May 2015.
